# Speak Gently and Kindly

**Published:** 1854-10

**Authors:** 


### SPEAK GENTLY AND KINDLY.

Speak gently, lady reader, for you may have a frail young creature in your employ, and she may be an orphan too—and not at all prepossessing in her manners—and sometimes disagreeable; and often do things to displease you; but will she be any the better, or you the happier, for speaking to her harshly or unkindly? She may have grown up without a mother's advice, or a father's care—no home but that accorded to her by strangers—no sister to share her griefs—no brother to shield her from the life-blasts of adversity—wandering from place to place, and no one to take an interest in her welfare as regards this life, or the one which is to come. She may feel lonely and forlorn, for there is nothing in the past for her to look back to with a pleasing recollection. The present is cheerless; the future dark and gloomy; and it is for you to be her friend, and help her through the toils of the day by speaking gently and kindly to her; and opening to her the avenues to your heart; and let your kindness be an oasis in the desert of her life, and teach her how to walk in wisdom's ways. Then she will be better qualified to take such course as will meet your approbation. But remember to speak always gently and kindly.

You may have a son, whom you once thought would be a comfort and a support in your declining years—but he has wandered far from the path of rectitude, and disappointed your most sanguine hopes, for he now prefers the grog-shop, or club-room, to the home-roof that would have sheltered him from the storms of life. Though he is in the downward road to ruin, remember, if you would win him back to honor's path, to speak gently and kindly to him. For where is that son, that has a heart, that can long be steeled to a mother's tears and kindness? A soft word, a friendly look, will vibrate every nerve of his heart. Then is the time to entwine the cords of tenderness around him, and draw him towards his once loved home. Then speak gently and kindly, for may be he will yet become the solace and staff of your journey to the tomb—but speak gently and kindly.

You may have an erring neighbor, that often gets into a passion, and will say and do things that astonishes you, but if you would convince him of his wrong, speak to him gently and kindly. He may sometimes say and do that which will injure your prosperity and reputation, but if you would control him, speak to him gently and kindly. "Be not overcome of evil, but overcome evil with good," is the command of inspiration. MAHALA.

*Griswold's Mills.*

---

AN AFFLICTED MAN.—A few days ago, a flying bookseller and stationer called at a publican's house in the village of——, and offered the landlord to sell him a copy of the "Afflicted Man's Companion." The landlord looked at it, and returned the copy, saying that he had a much better one already. The bookseller said he should like to see it. Come this way, said the publican, and, introducing him into a small parlor, showed the bookseller his dear wife lying on the floor dead drunk, with an empty whisky bottle by her side. There, said the publican, is my copy of the "Afflicted Man's Companion."

